# Bedaquiline and clofazimine resistance in *Mycobacterium tuberculosis*: an in-vitro and in-silico data analysis

**DOI:** 10.1016/S2666-5247(23)00002-2

**Published:** 2023-05

**Authors:** Lindsay Sonnenkalb, Joshua James Carter, Andrea Spitaleri, Zamin Iqbal, Martin Hunt, Kerri Marie Malone, Christian Utpatel, Daniela Maria Cirillo, Camilla Rodrigues, Kayzad Soli Nilgiriwala, Philip William Fowler, Matthias Merker, Stefan Niemann, Comprehensive Resistance Prediction for Tuberculosis: an International Consortium, Ivan Barilar, Simone Battaglia, Emanuele Borroni, Angela Pires Brandao, Alice Brankin, Andrea Maurizio Cabibbe, Joshua Carter, Daniela Maria Cirillo, Pauline Claxton, David A Clifton, Ted Cohen, Jorge Coronel, Derrick W Crook, Viola Dreyer, Sarah G Earle, Vincent Escuyer, Lucilaine Ferrazoli, Philip W Fowler, George Fu Gao, Jennifer Gardy, Saheer Gharbia, Kelen Teixeira Ghisi, Arash Ghodousi, Ana Luíza Gibertoni Cruz, Louis Grandjean, Clara Grazian, Ramona Groenheit, Jennifer L Guthrie, Wencong He, Harald Hoffmann, Sarah J Hoosdally, Martin Hunt, Zamin Iqbal, Nazir Ahmed Ismail, Lisa Jarrett, Lavania Joseph, Ruwen Jou, Priti Kambli, Rukhsar Khot, Jeff Knaggs, Anastasia Koch, Donna Kohlerschmidt, Samaneh Kouchaki, Alexander S Lachapelle, Ajit Lalvani, Simon Grandjean Lapierre, Ian F Laurenson, Brice Letcher, Wan-Hsuan Lin, Chunfa Liu, Dongxin Liu, Kerri M Malone, Ayan Mandal, Mikael Mansjö, Daniela Matias, Graeme Meintjes, Flávia de Freitas Mendes, Matthias Merker, Marina Mihalic, James Millard, Paolo Miotto, Nerges Mistry, David Moore, Kimberlee A Musser, Dumisani Ngcamu, Ngoc Nhung Hoang, Stefan Niemann, Kayzad Soli Nilgiriwala, Camus Nimmo, Nana Okozi, Rosangela Siqueira Oliveira, Shaheed Vally Omar, Nicholas Paton, Timothy EA Peto, Juliana Maira Watanabe Pinhata, Sara Plesnik, Zully M Puyen, Marie Sylvianne Rabodoarivelo, Niaina Rakotosamimanana, Paola MV Rancoita, Priti Rathod, Gillian Rodger, Camilla Rodrigues, Timothy C Rodwell, Eaysha Roohi, David Santos-Lazaro, Sanchi Shah, Thomas Andreas Kohl, Grace Smith, Walter Solano, Andrea Spitaleri, Philip Supply, Utkarsha Surve, Sabira Tahseen, Nguyen Thuy Thuong Thuong, Guy Thwaites, Katharina Todt, Alberto Trovato, Christian Utpatel, Annelies Van Rie, Srinivasan Vijay, Timothy M Walker, Sarah A Walker, Robin Warren, Jim Werngren, Maria Wijkander, Robert J Wilkinson, Daniel J Wilson, Penelope Wintringer, Xin Xiao Yu, Yang Yang, Yanlin Zhao, Shen-Yuan Yao, Baoli Zhu

**Affiliations:** a Molecular and Experimental Mycobacteriology, Research Center Borstel Leibniz Lung Center, Borstel, Germany; b Evolution of the Resistome, Research Center Borstel Leibniz Lung Center, Borstel, Germany; c National Reference Center, Research Center Borstel Leibniz Lung Center, Borstel, Germany; d Medical Scientist Training Program, Stanford University, Stanford, CA, USA; e Emerging Bacterial Pathogens Unit, Division of Immunology, Transplantation and Infectious Diseases, IRCCS San Raffaele Scientific Institute, Milan, Italy; f Center for Omics Sciences, IRCCS San Raffaele Scientific Institute, Milan, Italy; g Vita-Salute San Raffaele University, Milan, Italy; h European Bioinformatics Institute, Cambridge, UK; i Nuffield Department of Medicine, University of Oxford, Oxford, UK; j Department of Microbiology, P D Hinduja National Hospital and Medical Research Centre, Mumbai, India; k Tuberculosis Department, The Foundation for Medical Research, Mumbai, India; l National Institute of Health Research Oxford Biomedical Research Centre, John Radcliffe Hospital, Oxford, UK; m German Centre for Infection Research, Partner Site Hamburg-Lübeck-Borstel-Riems, Borstel, Germany

## Abstract

**Background:**

Bedaquiline is a core drug for the treatment of multidrug-resistant tuberculosis; however, the understanding of resistance mechanisms is poor, which is hampering rapid molecular diagnostics. Some bedaquiline-resistant mutants are also cross-resistant to clofazimine. To decipher bedaquiline and clofazimine resistance determinants, we combined experimental evolution, protein modelling, genome sequencing, and phenotypic data.

**Methods:**

For this in-vitro and in-silico data analysis, we used a novel in-vitro evolutionary model using subinhibitory drug concentrations to select bedaquiline-resistant and clofazimine-resistant mutants. We determined bedaquiline and clofazimine minimum inhibitory concentrations and did Illumina and PacBio sequencing to characterise selected mutants and establish a mutation catalogue. This catalogue also includes phenotypic and genotypic data of a global collection of more than 14 000 clinical *Mycobacterium tuberculosis* complex isolates, and publicly available data. We investigated variants implicated in bedaquiline resistance by protein modelling and dynamic simulations.

**Findings:**

We discerned 265 genomic variants implicated in bedaquiline resistance, with 250 (94%) variants affecting the transcriptional repressor (Rv0678) of the MmpS5–MmpL5 efflux system. We identified 40 new variants in vitro, and a new bedaquiline resistance mechanism caused by a large-scale genomic rearrangement. Additionally, we identified in vitro 15 (7%) of 208 mutations found in clinical bedaquiline-resistant isolates. From our in-vitro work, we detected 14 (16%) of 88 mutations so far identified as being associated with clofazimine resistance and also seen in clinically resistant strains, and catalogued 35 new mutations. Structural modelling of *Rv0678* showed four major mechanisms of bedaquiline resistance: impaired DNA binding, reduction in protein stability, disruption of protein dimerisation, and alteration in affinity for its fatty acid ligand.

**Interpretation:**

Our findings advance the understanding of drug resistance mechanisms in *M tuberculosis* complex strains. We have established an extended mutation catalogue, comprising variants implicated in resistance and susceptibility to bedaquiline and clofazimine. Our data emphasise that genotypic testing can delineate clinical isolates with borderline phenotypes, which is essential for the design of effective treatments.

**Funding:**

Leibniz ScienceCampus Evolutionary Medicine of the Lung, Deutsche Forschungsgemeinschaft, Research Training Group 2501 TransEvo, Rhodes Trust, Stanford University Medical Scientist Training Program, National Institute for Health and Care Research Oxford Biomedical Research Centre, Oxford University Hospitals NHS Foundation Trust, Bill & Melinda Gates Foundation, Wellcome Trust, and Marie Skłodowska-Curie Actions.

## Introduction

Multidrug-resistant (MDR) *Mycobacterium tuberculosis* complex strains, defined as strains resistant to at least isoniazid and rifampicin, are a serious challenge for global control of tuberculosis.^1^, ^2^ According to the 2022 WHO report, the incidence of drug-resistant tuberculosis increased between 2020 and 2021, with an estimated 450 000 new cases of rifampicin-resistant tuberculosis globally.^1^ New treatment regimens for MDR tuberculosis have been recommended that include the diarylquinoline drug bedaquiline.^3^ Bedaquiline has become central to MDR tuberculosis therapy, and also part of the shorter, all-oral regimen.^3^, ^4^

Resistance to bedaquiline has been associated with variants in the genes *atpE*,^5^, ^6^ *pepQ*,^6^ *Rv1979c*,^6^ and *Rv0678*.^6^, ^7^ *Rv0678* codes for a transcriptional inhibitor of *mmpS5–mmpL5* (encoding the MmpS5–MmpL5 efflux pump) by binding the upstream DNA region of these genes.^8^ Therefore, any modifications to *Rv0678* that reduce functionality will result in upregulation of the pump, inducing an elevated minimum inhibitory concentration (MIC) to bedaquiline.^9^, ^10^ Variants in *Rv0678* are the most common resistance mechanism against bedaquiline and also confer cross-resistance to the repurposed tuberculosis drug clofazimine.^6^, ^7^, ^8^, ^10^ Nevertheless, a comprehensive understanding of the associations between *Rv0678* variants and their resulting phenotype, as well as their structural effects on the transcriptional repressor Rv0678, is lacking.

In this study, we used in-vitro and in-silico approaches to address this problem, with the final aim of developing a comprehensive mutation catalogue for genotypic bedaquiline (and clofazimine) drug susceptibility testing to guide bedaquiline-containing MDR tuberculosis treatments.

**Research in context**

**Evidence before this study**

We searched PubMed, Google, and Google Scholar for articles published from Jan 1, 2014, to July 20, 2022, using the terms “TB”, “*Mycobacterium tuberculosis*”, “MTB”, “bedaquiline”, “clofazimine”, “treatment”, “clinical report”, “patient”, “MDR-TB”, “XDR-TB”, “diarylquinoline”, and “drug resistance”. Antibiotic resistance against bedaquiline has emerged as a substantial threat for the treatment of multidrug-resistant (MDR) tuberculosis. Variants in the gene *Rv0678*, which encodes a transcriptional regulator of the MmpS5–MmpL5 efflux pump, have been identified to confer resistance against bedaquiline and clofazimine. However, data on the correlation between phenotype and genotype are sparse, thus hampering rapid diagnostics and jeopardising new bedaquiline-based MDR tuberculosis regimens. In 2021, WHO released their first mutation catalogue for molecular diagnosis of drug-resistant tuberculosis, which did not feature variants implicated in resistance to bedaquiline. This lack of data was due to the low number of bedaquiline-resistant clinical isolates, and the difficulty in distinguishing *Rv0678* mutants and wild-type strains in phenotypic antimicrobial susceptibility testing.

**Added value of this study**

We first developed a new in-vitro evolution model resulting in the enrichment of a genetically diverse population of bedaquiline-resistant *Mycobacterium tuberculosis* complex mutants through long-term, sublethal drug exposure. Selected clones were further investigated for newly acquired variants and associated minimum inhibitory concentration, also revealing variants that confer cross-resistance against clofazimine. Using this model, we showed that bedaquiline dosages lower than the critical concentration can select for resistant mutants, a finding with wide-ranging consequences for the use of the drug in clinical settings. We compiled a comprehensive mutation catalogue, which included 265 variants implicated in resistance and susceptibility against bedaquiline, and the reclassification of several variants as benign (phylogenetic markers). Our in-vitro experiments also identified a large-scale genomic rearrangement as a new resistance mechanism for *M tuberculosis* complex strains. We compiled this catalogue from our in-vitro work, the phenotypic and genotypic data of a clinical strain collection of more than 14 000 isolates provided by the Comprehensive Resistance Prediction for Tuberculosis: an International Consortium project, and publicly available data. Our protein modelling showed different bedaquiline resistance mechanisms affecting Rv0678-mediated efflux pump regulation, including impaired DNA binding, reduction in protein stability, disruption of protein dimerisation, and alteration in affinity for its fatty acid ligand.

**Implications of all the available evidence**

This work has identified new bedaquiline resistance-mediating variants and provides a comprehensive mutation catalogue for molecular diagnostics. Our in-vitro evolution model can be used to describe resistance-associated variants before they arise in clinical strains. For the first time, we observed a spontaneous gene rearrangement event in vitro, which resulted in phenotypic resistance against bedaquiline, and which might be a new resistance mechanism for other antibiotics.

## Methods

### Study design

In this study, we established and applied an in-vitro evolution model that evolves *M tuberculosis* under subinhibitory drug concentrations. Such sublethal concentrations select for drug-resistant mutants in other bacterial species,^11^ and potentially mimic the physiological conditions in patients with tuberculosis, since bedaquiline does not sufficiently penetrate into necrotic lung lesions.^12^ We did a whole-genome sequencing analysis of in-vitro selected mutants and a large set of clinical strains collected by the Comprehensive Resistance Prediction for Tuberculosis: an International Consortium (CRyPTIC) project.^13^ Identified mutations were further investigated for putative effects on the structure and function of the Rv0678 protein.

Publicly available data (ie, in-vitro, in-vivo, and patient-derived strains) explicitly stating approval by an ethics committee were included in this study. Individual references are given in appendix 2. Approval for the use of clinical strains by the CRyPTIC study was obtained from the Taiwan Centers for Disease Control institutional review board (106209; Taipei City, Taiwan); University of KwaZulu-Natal biomedical research ethics committee (BE022/13; Durban, South Africa); University of Liverpool central university research ethics committees (2286; Liverpool, UK); institutional research ethics committee of The Foundation for Medical Research (FMR/IEC/TB/01a/2015 and FMR/IEC/TB/01b/2015; Mumbai, India); institutional review board of P D Hinduja Hospital and Medical Research Centre (915-15-CR; Mumbai, India); scientific committee of the Adolfo Lutz Institute (CTC-IAL 47-J/2017; São Paulo, Brazil); the ethics committee (81452517.1.0000.0059) and ethics committee review of Universidad Peruana Cayetano Heredia (Lima, Peru); and London School of Hygiene & Tropical Medicine (14924 /RR/10942; London, UK).

### In-vitro evolution experiments

In-vitro evolution experiments were done with the H37Rv reference strain (ATCC 27294). Bedaquiline was purchased from Janssen-Cilag (Neuss, Germany) and clofazimine from Sigma (C8895-1G; Darmstadt, Germany) and both were reconstituted from powder in dimethyl sulphoxide and stored at –20°C.

Bacteria were exposed in liquid culture to bedaquiline or clofazimine concentrations that were lower than the corresponding MICs, over five bacterial passages (appendix 1 p 12). The bacteria were plated on selective agar plates supplemented with the MIC of bedaquiline or clofazimine. Mutant colonies were selected from these plates for population sequencing and characterisation. Further details are given in appendix 1 (p 2).

### Whole-genome sequencing

DNA from in-vitro evolution experiments was isolated with cetyltrimethylammonium bromide, sequenced on the Illumina NextSeq500 platform (San Diego, CA, USA), and aligned to the reference genome (GenBank number NC_000962.3) with the MTBseq pipeline.^14^ We did long-read sequencing on the PacBio Sequel II System (Pacific Biosciences, Menlo Park, CA, USA; appendix 1 p 3).

### Screening of *Rv0678* variants in clinical strains

Clinical tuberculosis strains were collected by CRyPTIC partners in 27 countries worldwide and analysed in 14 different laboratories.^13^, ^15^ The full analysis pipeline for CRyPTIC is documented in appendix 1 (pp 3–4).^13^ Sample inclusion required MIC information for at least bedaquiline.

### Phenotyping

MICs for in-vitro single selected mutants were established by resazurin microtitre assay, and the BACTEC MGIT 960 SIRE Kits (Becton Dickinson, Franklin Lakes, NJ, USA). Each mutant was tested at least once with biological duplicates; MICs of independently selected mutants with the same mutation were pooled together and represented as a range; and six mutants were not tested against clofazimine because of culture issues (mutants would not grow again after storage). CRyPTIC strains were MIC-tested with a 96-well broth microdilution plate, as previously published.^15^ The plates were read manually by diagnostic laboratory scientists, by the automated reading software Automated Mycobacterial Growth Detection Algorithm,^16^ and by a citizen science project (BashTheBug) for additional verification.^17^ Procedural details of MIC testing are provided in appendix 1 (pp 4–5).

### *Rv0678* variant literature search

We did an extensive search to include and summarise previously published variants associated with resistance to bedaquiline or clofazimine, or both, from in-vitro, in-vivo, and clinical strains. We searched PubMed, Google, and Google Scholar for articles published from Jan 1, 2014, to July 20, 2022, using the terms “TB”, “*Mycobacterium tuberculosis*”, “MTB”, “bedaquiline”, “clofazimine”, “treatment”, “clinical report”, “patient”, “MDR-TB”, “XDR-TB”, “diarylquinoline”, and “drug resistance”.

### Data analysis and phenotypic interpretation

Because of the small sample size for most variants, we did not do a statistical review. Instead, the phenotype (ie, resistant, susceptible, borderline, and undetermined) for isolates with a single variant in a resistance-associated gene (ie, *Rv0678, atpE, pepQ*, or *Rv1979c*) was systematically identified on the basis of MIC values on different culture media; isolates that also harboured an *mmpL5* or *mmpS5* variant were represented separately (appendix 2). Interpretation of MICs was based on WHO critical concentrations, or based on individual studies, and details of the grading system are mentioned in appendix 1 (p 5).

Discrepancies between methods, studies, and conflicting phenotypes were catalogued as undetermined. We implemented the following confidence grading: high confidence (at least three strains with corresponding phenotypes); medium confidence (at least two strains with corresponding phenotypes and a wild-type ancestor); and low confidence (two or fewer strains with corresponding phenotypes).

### Structural modelling

An experimental structure of Rv0678 (Protein Data Bank number 4NB5) was visualised using UCSF Chimera (version 1.14).^18^ We did the structural alignment of the winged helix-turn-helix domain using the MatchMaker tool in Chimera with a Needleman–Wunsch algorithm using a BLOSUM62 matrix. We modelled all protein stabilities, and protein–protein and protein–DNA interactions using established mutation Cutoff Scanning Matrix (mCSM) methods.^19^ Further method details and statistics are given in appendix 1 (p 6).

### Molecular dynamics simulations

We prepared all the simulated systems using the BiKi Life Sciences software suite, version 1.3.5.^20^ For atoms less than 1·1 Å apart, we calculated electrostatic forces directly; for atoms further apart, we calculated electrostatic forces using the Particle Mesh Ewald method. To detect allosteric signal transmission, we calculated allosteric communication networks using the Pocketron module in the BiKi Life Sciences suite.^20^ Detailed methodology is discussed in appendix 1 (p 7).

### Role of the funding source

The funders of the study had no role in study design, data collection, data analysis, data interpretation, or writing of the report.

## Results

We first developed an in-vitro evolution model, which evolves *M tuberculosis* under a weak drug selection pressure, during an extended period (appendix 1 p 12). Exposure to bedaquiline or clofazimine at concentrations as low as an eighth of the wild-type MIC, during 20 days (12–19 generations), led to the selection and enrichment of significantly more resistant clones compared with growth in the absence of the drug (appendix 1 pp 13–14). As expected, enrichment of resistant populations was reduced at early timepoints (eg, after 4 days). These findings indicate that resistant mutants are selected at concentrations far lower than the critical concentrations of these drugs.

From the bedaquiline-supplemented and clofazimine-supplemented agar plates, we then randomly selected 270 single bacterial colonies (appendix 1 p 12). 214 mutants were successfully phenotyped and genotyped. All mutants exhibited an elevated bedaquiline MIC compared with the drug-susceptible ancestor in the resazurin microtitre assay. 183 (86%) of the 214 mutants had a variant in *Rv0678*, 18 (8%) had a variant in *atpE*, and 13 (6%) did not have any variant in a known resistance-associated gene (table).

**Table:** In-vitro selected variants in *Rv0678, atpE*, and other genes conferring resistance to bedaquiline and clofazimine

		**Mutation**	**Number of times selected**	**Bedaquiline MIC (mg/L)**	**Bedaquiline MIC fold increase**	**Clofazimine MIC (mg/L)**	**Clofazimine MIC fold increase**
Wild-type ancestor			..	0·25 to 0·50	..	0·5 to 1·0	..
*Rv0678*							
	Promotor	−10_–9insG	1	2	4 to 8	1	1 to 2
	6fs*	16_17delG	1	1	2 to 4	2	2 to 4
	9fs*	26_27delAG	1	2	4 to 8	NA	NA
	14fs*	40_41delC	1	2	4 to 8	2	2 to 4
	17fs	49_50delA	1	2	4 to 8	2	2 to 4
	21fs	62_63delA	1	2	4 to 8	1	1 to 2
	Gly24Val*	71G→T	3	2	4 to 8	2	2 to 4
	Gly25Ser	73G→A	1	2	4 to 8	NA	NA
	30fs	89_90delG	1	2	4 to 8	2	2 to 4
	Arg38Ter	112C→T	4	2	4 to 8	2	2 to 4
	Arg38Pro	113G→C	1	1	2 to 4	2	2 to 4
	Leu40Ser	119T→C	1	1	2 to 4	2	2 to 4
	40fs	120_121delG	1	1	2 to 4	2	2 to 4
	Leu43Pro	128T→C	5	1 to 2	2 to 8	2	2 to 4
	Leu43Arg	128T→G	1	1	2 to 4	2	2 to 4
	Leu44Pro*	131T→C	4	1	2 to 4	2	2 to 4
	Arg50Trp	148C→T	16	1	2 to 4	2	2 to 4
	Gln51Arg	152A→G	2	2	4 to 8	4	4 to 8
	Glu54Ter	160G→T	1	2	4 to 8	2	2 to 4
	Glu55Ter*	163G→T	1	1	2 to 4	2	2 to 4
	Ala57Glu	170C→A	1	2	4 to 8	2	2 to 4
	Ala62Asp*	185C→A	1	2	4 to 8	2	2 to 4
	Ser63Gly	187A→G	3	2	4 to 8	2	2 to 4
	64fs*	192_193insG	18	1 to 4	2 to 16	2 to 4	2 to 8
	65fs*	193_194delG	13	1 to 2	2 to 8	2 to 4	2 to 8
	Gly66Glu	197G→A	1	2	4 to 8	1	1 to 2
	Ser68Gly*	202A→G	9	1 to 2	2 to 8	2	2 to 4
	Arg72Trp	214C→T	2	1 to 2	2 to 8	2	2 to 4
	Arg72Leu	215G→T	3	2 to 4	4 to 16	4 to 6	4 to 12
	Leu74Pro*	221T→C	8	1 to 2	2 to 8	2 to 4	2 to 8
	76fs*	228_229insT	1	2	4 to 8	NA	NA
	Phe79Val	235T→G	1	1	2 to 4	2	2 to 4
	Phe79Cys	236T→G	2	1	2 to 4	2	2 to 4
	Ala84Val	251C→T	2	1 to 2	2 to 8	1 to 2	1 to 4
	88fs	263_264insT	1	2	4 to 8	1	1 to 2
	92fs	274_275insA	3	1	2 to 4	2	2 to 4
	94fs	281_282delG	1	2	4 to 8	2	2 to 4
	97fs	291_292insA	2	2	4 to 8	2	2 to 4
	98fs	292_293delA	1	2	4 to 8	2	2 to 4
	Ala99Val*	296C→T	17	1 to 2	2 to 8	1 to 2	1 to 4
	Ala102Thr*	304G→A	4	1 to 4	2 to 16	2 to 4	2 to 8
	Gly103Arg	307G→C	1	1	2 to 4	2	2 to 4
	Leu114Pro	341T→C	1	2	4 to 8	2	2 to 4
	Gln115Ter	343C→T	1	2	4 to 8	2	2 to 4
	Ala118Asp*	353C→A	1	1	2 to 4	2	2 to 4
	120fs*	360_361delG	8	1 to 4	2 to 16	2 to 4	2 to 8
	Gly121Glu	362G→A	3	2	4 to 8	2 to 4	2 to 8
	Leu122Pro	365T→C	1	2	4 to 8	2	2 to 4
	125fs	374_375delT	1	2	4 to 8	2	2 to 4
	Leu125Pro	374T→C	1	2	4 to 8	2	2 to 4
	Arg132Pro	395G→C	1	2	4 to 8	2	2 to 4
	Arg135Trp	403C→T	1	2	4 to 8	2	2 to 4
	Leu136Pro	407T→C	2	2	4 to 8	2 to 4	2 to 8
	139fs	417_418insCGGGATCTGTTGGCATATAT	3	1 to 2	2 to 8	2	2 to 4
	142fs	425_426delT	1	2	4 to 8	2	2 to 4
	142fs	426_427insTTGGCATA	1	2	4 to 8	NA	NA
	Tyr145Ter*	435T→G	6	1 to 2	2 to 8	2	2 to 4
	Ser151Pro	451T→C	1	2	4 to 8	2	2 to 4
	Leu154Pro	461T→C	2	2	4 to 8	2	2 to 4
	155fs	465_466insC	2	1 to 2	2 to 8	2	2 to 4
	Arg156Ter	466C→T	3	2	4 to 8	2	2 to 4
*atpE*							
	Asp28Gly	83A→G	1	≥10	≥20	NA	NA
	Asp28Val	83A→T	3	≥8	≥16	NA	NA
	Glu61Asp*	183G→C	4	2 to ≥10	4 to ≥20	1	1 to 2
	Glu61Asp	183G→T	3	≥10	≥20	1 to 2	1 to 4
	Ala63Pro*	187G→C	7	≥10	≥20	1 to 2	1 to 4
Other		..	13	2	4 to 8	1 to 2	1 to 4

The genotype of each variant was identified by whole-genome sequencing. The 13 mutants under the category Other do not have any variant in a resistance-associated gene. MIC was established by resazurin microtitre assay and compared with the drug-susceptible wild-type ancestor to describe MIC fold increase. Each single mutant was tested at least one time with biological duplicates. MICs of independently selected mutants with the same mutation were pooled together and represented as a range. Additional details including variant position, selection drug, and selection concentration are included in appendix 3 (p 1). fs=frameshift. MIC=minimum inhibitory concentration. NA=not available.

* One or more selected mutants harbour a variant in a second gene; further details are given in appendix 3 (p 4).

Compared with the wild type, mutants that harboured a variant in *Rv0678* had a 2–16-fold MIC increase to bedaquiline, whereas variants in *atpE* exhibited a fold MIC increase of 4–20 or more (table). For 12 randomly selected mutants with seven different variants, we verified the resistant phenotype using the BACTEC MGIT assay (appendix 3 p 2).

Among the 214 bedaquiline-resistant mutants, we found 61 variants in *Rv0678* affecting 54 different codons (table), of which 22 (36%) were frameshift variants, 33 (54%) led to a non-synonymous amino acid variant, and six (10%) prematurely inserted stop codons.

In addition to single colony sequencing, we also used a more unbiased approach based on population sequencing of all the mutant colonies on a given selection plate. We identified 45 additional variants in *Rv0678*, one in *atpE* (Ala63Thr), and four in *pepQ* (*Rv2535c*; Phe97Val, Gly96Gly, Val92Gly, and Ala87Gly; appendix 3 p 3).

No mutant had more than one resistance-conferring variant in *Rv0678* or *atpE*; however, 38 mutants harboured a second variant in one of 14 genes that have not been linked to resistance (appendix 3 p 4). The most common off-target variants found were Arg119His in *Rv1890c* (11 mutants) and Ter130Arg in *Rv1871c* (seven mutants); both genes encode conserved hypothetical proteins. Other secondary variants occurred in genes that were involved in cell wall synthesis, information pathways, metabolism and respiration, protein regulation, or lipid metabolism (appendix 3 p 4).

**Figure 1:**
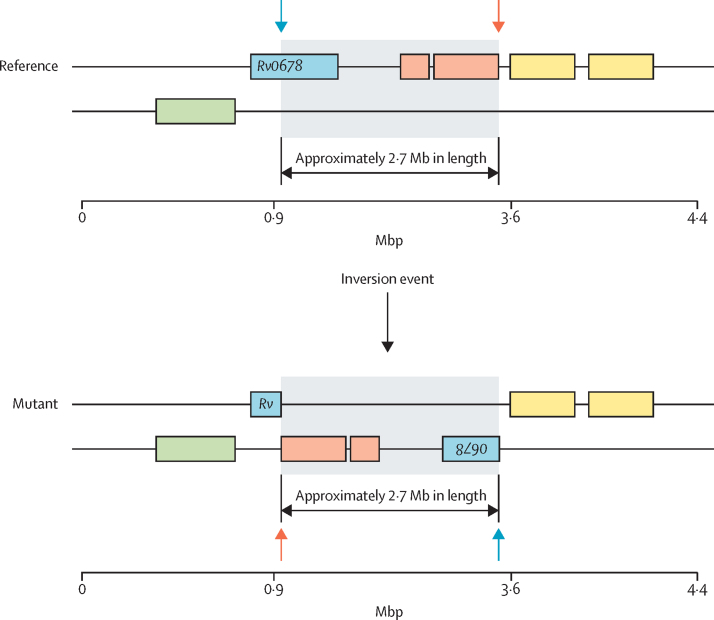
Large-scale gene rearrangement in the *Rv0678* gene De-novo assembly with the PacBio SMRT Link software indicated a 2·7 Mb inversion (grey box) affecting Rv0678 (ie, splitting Rv0678 into halves; blue box)*,* at positions 779 073 (coding sequence; amino acid 28 in Rv0678; marked by a blue arrow) and 3 552 584 (intergenic region; marked by an orange arrow), flanked by transposase related genes (orange and yellow boxes).

13 mutants selected after the in-vitro evolution experiments were phenotypically resistant to bedaquiline but did not have a variant in any bedaquiline resistance-associated genes. We randomly selected three of these mutants for long-read sequencing with the PacBio Sequel II System and did de-novo assemblies. All three assemblies showed the same large-scale genome rearrangement in which a 2·7 Mb fragment was inverted (figure 1). This genomic rearrangement split the *Rv0678* gene into halves, which was not detected by a classic reference mapping of short reads.

We aimed to comprehensively describe variants implicated in bedaquiline resistance and susceptibility in clinical strains to supplement our in-vitro mutation catalogue. We initially analysed the phenotypes and genotypes of more than 14 000 new clinical tuberculosis strains collected by the CRyPTIC Consortium. This dataset contained more than 1200 clinical strains harbouring variants in bedaquiline or clofazimine resistance-associated genes—*Rv0678, atpE, Rv1979c*, or *pepQ*. We also did a literature search to include published strains from other clinical reports plus in-vitro and in-vivo derived mutants. In total, we included 1918 strains in the final analysis, with a spectrum of bedaquiline-susceptible phenotypes (appendix 2).

In these strains, we identified 329 unique variants in the *Rv0678* gene, 250 of which were associated with a resistant or borderline bedaquiline phenotype; of these 250 variants, 117 were also deemed cross-resistant against clofazimine. Because of the large diversity of variants and differences in the interpretations of resistance and susceptibility in different culture media, we included a confidence scoring (high, medium, and low) for each variant (appendix 2). We further catalogued 48 variants with a susceptible phenotype and 31 variants associated with a large range of phenotypes, which were classified as undetermined. We additionally screened for co-occurring variants in the efflux pump genes *mmpL5* and *mmpS5*, because variants in these genes have an epistatic link in reverting an *Rv0678*-mediated resistance phenotype back to susceptible.^21^ In the CRyPTIC dataset, we found 19 isolates with variants in *Rv0678* plus *mmpS5* or *mmpL5*, nine of which occurred in phenotypically resistant strains.

Of 208 mutations found in bedaquiline-borderline or bedaquiline-resistant clinical isolates, we confirmed 15 (7%) with our in-vitro evolution model (appendix 2). Conversely, 15 (22%) of 67 mutations selected in vitro were previously found in bedaquiline-borderline and bedaquiline-resistant clinical isolates.

**Figure 2:**
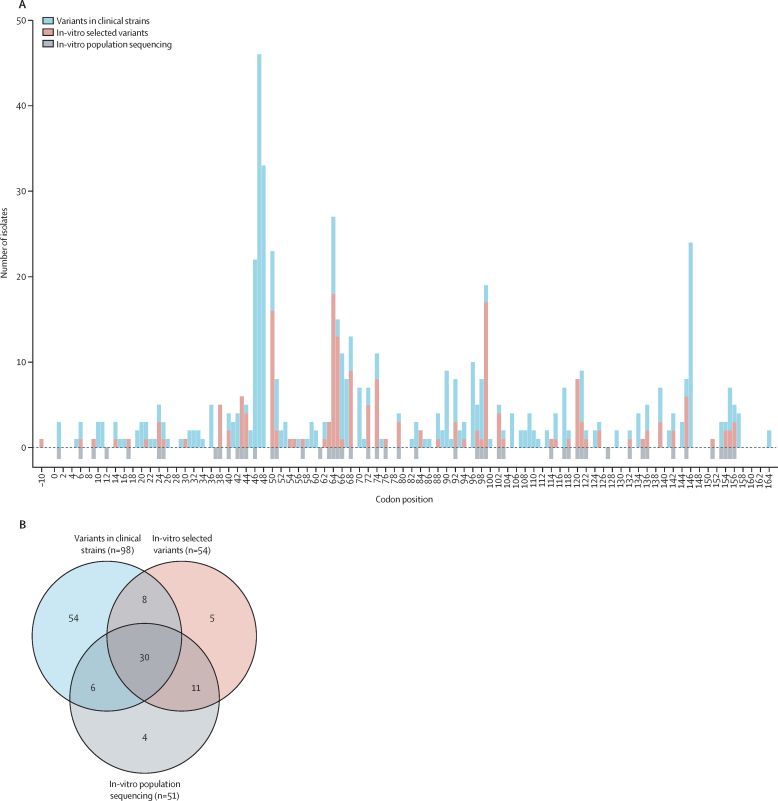
Affected codon positions in *Rv0678* leading to bedaquiline resistance in this study and in clinical strains (A) The codon positions in *Rv0678* that conferred bedaquiline resistance (and borderline resistance) were compared between three datasets: in-vitro selected mutants, which were single clones isolated from in-vitro evolution experiments (appendix 3 [p 1]); in-vitro population sequencing, corresponding to variants detected from deep sequencing of the entire mutant population in in-vitro evolution experiments (appendix 3 [p 2]); and variants in clinical strains from the CRyPTIC project and literature-reported strains (appendix 2). The variant at base-pair position –10 is the variant in the promoter region. In-vitro population sequencing was not quantifiable by number of isolates, hence the negative bar orientation for the affected codon positions. (B) The Venn diagram shows the number of overlapping codon positions where a mutation was detected between the three datasets. CRyPTIC=Comprehensive Resistance Prediction for Tuberculosis: an International Consortium.

Resistance-associated variants were scattered over the entire sequence of *Rv0678* and we did not identify any dominant cluster (figure 2). When comparing affected codon positions between our in-vitro work and resistant clinical strains (ie, CRyPTIC and published studies), 118 codons were affected throughout *Rv0678*. In this comparison, 44 (69%) of 64 coding positions detected in in-vitro selected isolates were also found in clinical strains, and 44 (45%) of 98 codon positions identified in clinical strains were recovered in our in-vitro work. In the *atpE* gene, we identified 11 variants implicated in bedaquiline resistance and one linked to bedaquiline susceptibility. Additionally, we found 17 variants in *Rv1979c* and *pepQ* genes, of which only four were linked with bedaquiline resistance. By cross-referencing multiple studies and considering the abundance of strains harbouring these variants, we could conclude that seven of these previously described *Rv1979c* and *pepQ* variants were phylogenetic markers, and probably benign.^22^

To improve our mechanistic understanding of how *Rv0678* variants could lead to bedaquiline or clofazimine resistance, we mapped missense variants (ie, an alternative amino acid substitution) from our catalogue to a previously established experimental structure of Rv0678 (appendix 2).^23^ Because variants in different parts of the protein structure are likely to have different effects, we investigated four different resistance mechanisms: reducing protein stability, impairing DNA binding, altering protein dimerisation, and reducing affinity for the fatty acid ligand. The numerous introductions of premature stop codons and frameshift variants suggest loss of functional Rv0678 protein is a common mechanism of resistance.^6^, ^7^ Consistently with this mechanism, we identified significant differences in the predicted effects of resistant versus susceptible missense variants on protein stability (Fisher's exact test p=0·0071) and SNAP2 scores (a proxy measure for deleteriousness of variants; Wilcoxon test p=0·0021; appendix 3 pp 5–6).

**Figure 3:**
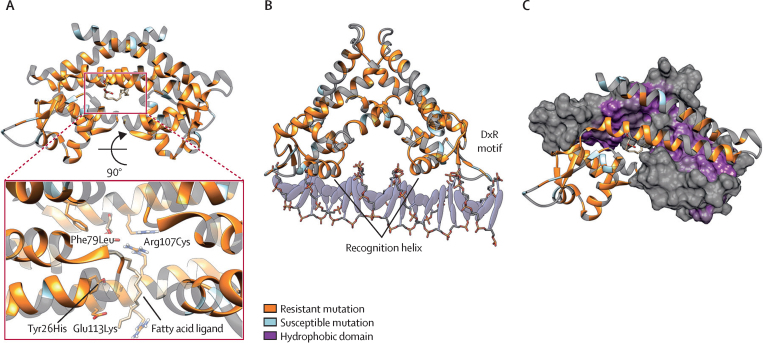
Structural mechanisms of bedaquiline resistance (A) Rv0678 dimer with resistant (orange) and susceptible (blue) mutations shown. The pullout highlights the resistant mutations in the ligand binding pocket. (B) Rv0678 dimer modelled onto DNA, with conserved multiple antibiotic resistance regulator (MarR) family DNA binding elements. (C) Resistant mutations occur across the hydrophobic dimerisation domain.

Because of the absence of an experimentally established DNA-bound structure of Rv0678, to understand how variants could affect DNA binding we structurally aligned the winged helix-turn-helix DNA binding domain of Rv0678 to several other transcription factors within the multiple antibiotic resistance regulator (MarR) family, whose DNA-bound structures have been identified (figure 3B; appendix 1 p 14).^24^ This homology model highlighted that variants clustered around amino acids 62–68 are associated with the highly conserved recognition α-helix and variants clustered around amino acids 88–92 are associated with the conserved DxR motif, which directly bind the DNA (figure 3B; appendix 3 p 5).^24^

We also investigated the possibility that variants alter Rv0678 function through changes in protein–protein dimerisation. When Rv0678 is not bound to DNA, variants at the dimer interface were often found in resistant strains (27 of 29 interface variants linked to bedaquiline resistance), suggesting they might affect dimer formation or stability, or both (figure 3C; appendix 3 p 5). However, computational prediction of interface effects of variants on non-DNA-bound dimer stability showed no difference in destabilisation as defined by mCSM protein–protein interface ΔΔG ≤–1 kcal/mol (ΔΔG being the changes in binding affinity due to mutation; 16 of 27 bedaquiline-resistant variants, one of two bedaquiline-susceptible variants). Interpretation of the DNA-bound dimer interface was not feasible because of the substantial conformational rearrangement of the dimer α-helices upon DNA binding (appendix 1 p 14). All strains with a variant in an amino acid that interact with the fatty acid ligand 2-stearoylglycerol were bedaquiline-resistant. 2-stearoylglycerol, a fatty acid glycerol ester, is the probable natural substrate of the Rv0678 transcriptional regulator, which is necessary for the dimerisation of the Rv0678 homodimers. Therefore, bedaquiline-resistant variants in this region suggest that disruption of ligand binding results in loss of DNA binding and confers resistance (figure 3A). This association remains to be biochemically investigated.

**Figure 4:**
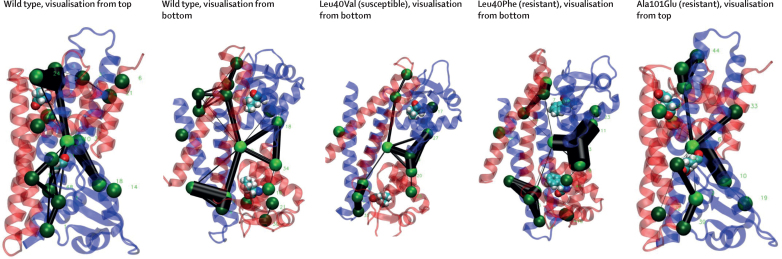
Networks of the most persistent pockets found in the wild-type and mutated Rv0678 The pocket crosstalk analysis on simulated systems identifies allosteric signalling, by measuring the exchange of atoms between adjacent pockets. Each pocket (ie, network node) is represented as a green sphere, and pockets are connected via network edges (black lines). The width of each network edge is proportional to the communication frequency. Therefore, the larger the network edge, the more frequently atoms are exchanged between the two pockets indicating a strong dynamic connection. All systems shared a large common pocket located at the interface of the two monomers (appendix 1 [p 17]). Comparative analysis showed that the resistance-conferring mutations, Leu40Phe and Ala101Glu, displayed different network edges during the 100 ns simulations with respect to the wild type, whereas Leu40Val recapitulates the main interaction network as found in the wild type.

Rv0678 needs to undergo a conformational rearrangement to bind DNA, which is not captured by the static structures described in the previous section. We used molecular dynamics simulations to understand how particular variants in the hinge region (connecting the DNA binding and dimerisation domains) might disrupt this motion and lead to resistance. We did 100 ns simulations on wild-type Rv0678 and on mutations with Leu40Phe or Ala101Glu variants (resistance-associated) or the Leu40Val variant (consistently phenotypically susceptible), which are all positioned in the hinge region and do not affect DNA binding or protein folding. Using a pocket crosstalk network analysis approach, we analysed the exchange of atoms between neighbouring pockets in the simulated trajectories for each of the variants, where an altered exchange of atoms (network edges) implies a change to the conformational flexibility or allosteric signalling, or both, of the protein. We found that variants from resistant strains (Leu40Phe and Ala101Glu) changed the conformational dynamics and pocket crosstalk of the individual Rv0678 monomers compared with the wild-type or the Leu40Val variant (figure 4). Ala101Glu formed a new stable salt-bridge interaction in the hinge domain, which could disrupt the conformational flexibility required by this region to transition between the DNA-bound and ligand-bound homodimer states (appendix 1 p 15).

## Discussion

In this study, we have identified new bedaquiline resistance determinants by combining an in-vitro evolution model with whole-genome sequencing analysis of more than 14 000 clinical strains, quantitative MIC data, and protein structural modelling. Collectively, this work extends our knowledge on resistance development in clinical tuberculosis strains and on the use of genomics in providing personalised medicine to patients with a highly resistant tuberculosis infection, through bolstering genotypic catalogues with a phenotypically verified variant list for bedaquiline (and clofazimine).

Although bedaquiline has been adopted worldwide as a core drug in treating MDR tuberculosis,^3^ antibiotic susceptibility testing for this compound is rarely available and patients are mainly treated empirically. Studies have reported unsuccessful treatments or poor outcomes, or both, in patients with MDR tuberculosis infection, as variants in *Rv0678* emerge that are associated with bedaquiline resistance.^9^, ^10^ Another difficulty in detecting bedaquiline resistance is that many resistant strains only exhibit a minor MIC increase, such that the epidemiological cutoff value is only just exceeded: this aspect is especially true for strains harbouring *Rv0678* variants. Such misdiagnosis can promote the selection and subsequent transmission of MDR tuberculosis strains. For example, in Eswatini and South Africa, patients infected with an MDR outbreak strain harbouring the rifampicin-resistant variant Ile491Phe in *rpoB* continued to receive rifampicin even though it was ineffective because this variant was not detectable by conventional phenotypic or genotypic testing.^25^

Producing a comprehensive variant list will aid in the diagnosis of bedaquiline-resistant tuberculosis infections. This work catalogues 265 unique genomic variants across four genes implicated in bedaquiline-resistant and bedaquiline-borderline phenotypes. Not all variants lead to resistance, as 53 variants appeared in bedaquiline-susceptible strains (with a further 32 variants being undetermined; appendix 2). Furthermore, in the clinical strains provided by the CRyPTIC Consortium, we found that nine of 48 bedaquiline-susceptible strains with *Rv0678* variants harboured an additional variant in the *mmpS5* or *mmpL5* efflux pump genes. These variants in *mmpS5* or *mmpL5* might have an epistatic link with *Rv0678* by abrogating the efflux pump, reconstituting the susceptible phenotype.^21^ This mechanism needs to be explored experimentally to understand the frequency, distribution, and origin of these inactivating variants and to elucidate their clinical relevance.

Importantly, in-vitro evolution experiments identified 67 resistance-conferring variants, 40 (60%) of which are new. Furthermore, this model selected 18 (27%) variants that overlapped with those found in clinical isolates, and 21 (31%) that had been previously selected in vitro (appendix 2). These results highlight the value of in-vitro evolution experiments as a complementary method for prospective identification of resistance, compared with traditional surveillance of clinical strains.

Additionally, we identified several off-target variants co-selected with the resistant variant; these variants can be further investigated for potential phenotypic effects (epistatic, compensatory, etc). By using PacBio sequencing to establish the full genome sequence of seemingly wild-type mutants, we were able to identify a large genomic inversion that disrupts *Rv0678,* which is a new bedaquiline resistance mechanism. Although this model did not abundantly overlap with the variation of resistance in clinical strains, it encompassed a wide diversity of variants and uncovered a new potential resistance mechanism through genomic rearrangements.

Another method for prospectively identifying variants that confer resistance is protein structure modelling, which has recently been combined with machine learning methods to design algorithms for predicting resistance to rifampicin, isoniazid, and pyrazinamide.^26^, ^27^, ^28^, ^29^ This study validated several resistance mechanisms in *Rv0678* with quantifiable structural effects (protein stability, dimer interactions, and interaction with the DNA), suggesting that a structure-based machine learning approach could also be successful for predicting bedaquiline resistance. Future work should explore the use of these features in a predictive model (eg, multivariable logistic regression, support vector machine, and neural networks) with an independent test set of variants for validation.

Of note, not all resistance-associated variants had clear effects on protein stability, dimer interactions, or DNA–ligand binding. In this Article, we used the pocket crosstalk approach, which monitors the exchange of atoms between adjacent pockets, and therefore characterises putative allosteric signalling transmission and conformational flexibility. Our analysis of the wild-type protein identified a common pocket located at the interface of the two monomers that communicates with other smaller pockets throughout the Rv0678 dimer (appendix 1 p 16). Our simulations showed that resistant variants (Leu40Phe and Ala101Glu) that do not have other apparent effects on protein stability or function cause distinct changes in the network edges compared with the wild-type and Leu40Val variants, possibly blocking or impairing the conformational change required to bind DNA.

Variants in *atpE* (coding for the target of bedaquiline) were rare among the in-vitro selection experiments and clinical strains. Besides variants in *Rv0678*, variants in *pepQ* and *Rv1979c* have also been linked to bedaquiline and clofazimine resistance, although these are rarely observed in clinical isolates and relevance remains unclear.^6^, ^10^ In this study, four variants could be linked to a resistant bedaquiline phenotype in these genes. Because we had such a large diverse collection of strains provided by the CRyPTIC Consortium, we further identified seven phylogenetic variants in these genes, which should be considered benign when identified in specific sublineages within lineage 2 or 4 (appendix 2).

This study had some limitations. External MIC data from previously published studies were collected with different phenotypic assays, some of which did not have WHO-endorsed critical concentrations. Thus, the interpretation of some variants in our catalogue might need to be updated upon revision or establishment of clinical breakpoints. In case of a wide MIC range for isolates with individual mutations or discrepancies in the interpretation between different phenotypic assays, we chose to interpret those mutations as undetermined.

In conclusion, our work advances the understanding of bedaquiline resistance. We established a comprehensive mutation catalogue, comprising variants and structural variations associated with bedaquiline (and clofazimine) resistance, benign variants, and variants implicated with susceptibility. This information can immediately be implemented in DNA sequencing-based diagnostic approaches such as the Deeplex Myc-TB assay, and existing bioinformatic pipelines for resistance prediction, or as a guidance for diagnostic laboratories and clinicians for the interpretation of molecular testing results. This work will facilitate the design of bedaquiline-containing MDR tuberculosis therapies.

## Data sharing

Genomes from in-vitro experiments can be downloaded from the European Nucleotide Archive (project number ERX5619327); PacBio sequenced accession numbers are ERX10074915, ERX10074916, and ERX10074917. Genomes for CRyPTIC strains are available at http://ftp.ebi.ac.uk/pub/databases/cryptic/release_june2022/, and can be accessed by following the instructions provided by Brankin and colleagues.^30^

## Declaration of interests

AS is the cofounder and owner of shares in BiKi Technology, which sells the BiKi Life Sciences software suite used in this study's analysis. PWF has received consulting fees from Global Pathogen Analysis Service. All other authors declare no competing interests.
