# Supplementary material for: Bedaquiline and clofazimine resistance in Mycobacterium tuberculosis: an in-vitro and in-silico data analysis
Source: Lancet Microbe. 2023 May;4(5):e358–68. doi: 10.1016/S2666-5247(23)00002-2 (PMC10156607; doi:10.1016/S2666-5247(23)00002-2)
Supplement: Supplementary appendix 1 [file mmc1.pdf]

THE LANCET
Microbe

Supplementary appendix 1

This appendix formed part of the original submission and has been peer reviewed. We post it as supplied by the authors.

Supplement to: Sonnenkalb L, Carter JJ, Spitaleri A, et al. Bedaquiline and clofazimine resistance in *Mycobacterium tuberculosis*: an in-vitro and in-silico data analysis. *Lancet Microbe* 2023; published online March 29. https://doi.org/10.1016/S2666-5247(23)00002-2.

Appendix 1- Supplementary Material

Contents

Materials and Methods 2

In vitro evolution experiments 2

Whole genome sequencing 3

Screening of Rv0678 mutations in clinical samples via the CRyPTIC strain collection 3

Phenotyping 4

Phenotypic interpretation 5

Rv0678 variant literature search 6

Structural modelling 6

Molecular dynamics simulations 7

Authors and members of the Comprehensive Resistance Prediction for Tuberculosis: an International Consortium 8

References 10

Supplemental Figures 12

Figure S1: *In vitro* evolutionary experimental design for resistance variant selection, detection and analysis. 12

Figure S2: Sub-lethal exposure of antibiotics enriches drug resistant populations in a dose dependent manor. 13

Figure S3: Rv0678 C-terminal domain undergoes conformational change upon DNA binding. 14

Figure S4. Zoom-in of the persistent interaction along the A101E simulations. 15

Figure S5: Localization of the main pockets in Rv0678 (4NB5), shown in blue surface. 16

Figure S6: Homodimer pocket communication. 17

Materials and Methods

In vitro evolution experiments

In vitro experiments were carried out with H37Rv strain (ATCC 27294). Bedaquiline was purchased from Janssen-Cilag GmbH and clofazimine was ordered from Sigma (C8895-1G) both were reconstituted from powder in DMSO and stored at -20°C.

Evolutionary experiments were conducted with the *Mycobacterium tuberculosis* complex (Mtbc) lab strain H37Rv. The bacteria were cultured from frozen stocks and (pre)cultured at 37° in 7H9 medium, supplemented with 0·2% glycerol, 10% oleic albumin dextrose catalase (OADC), and 0.05% Tween[80] (termed culture medium). At exponential growth phase (between 0·3 and 0·6 optical density (OD) of 600nm), bacteria were transferred to new culture medium with a final OD of 0.05 in 50 mL, additionally supplemented with either bedaquiline (BDQ) or clofazimine (CFZ). Four concentrations below the minimum inhibitory concentration (MIC) were included for each drug at 1:2, 1:4, 1:8, and 1:16 below the strain MIC (and an antibiotic-free control). The MIC of the susceptible WT ancestor was determined to be 0·12 mg/L BDQ and 0·5 mg/L CFZ in this culturing system.

These experiments were carried out over 20 days with five culture passages total. Culture passages were conducted every four days with a final culture OD of 0·05 transferred. After passages 1, 3, and 5; bacteria were plated on selective agar plates of 7H11, supplemented with 0·5% glycerol, 10% OADC and either 0·12 (1:2 MIC) or 0·25 (MIC) mg/L of BDQ, or 0·25 (1:2 MIC) mg/L CFZ. After 21-28 days of growth on selective agar plates, single colonies were transferred to culture medium. Single colonies were selected from multiple plates of variable sizes and morphologies then grown for 2-4 weeks in antibiotic free 7H9 broth. Two 1 mL aliquots were frozen at -80 for storage, and an additional bacterial sample was taken for whole genome sequencing (WGS). All remaining colonies (collected by experimental condition) were scaped from agar plates using sterile loops, and collected for WGS, i.e. total population sequencing. This population sequencing aimed to remove the bias of single colony selection and give an overview of all variants in the total mutant populations throughout resistance-associated genes.

Whole genome sequencing

Isolates from *in vitro* evolutionary experiments underwent DNA isolation by CTAB method.[1] Paired-end DNA library preparations and sequencing was performed with Illumina technology (Nextera-XT and NextSeq500) according to the manufacturer's instructions and with a minimum average genome coverage of 50x. Fastq files (raw sequencing data) were mapped to the *M. tuberculosis* H37Rv reference genome (GenBank ID: NC_000962.3) using the MTBseq pipeline.[2]

Briefly, for the analysis of single mutants we considered single nucleotide polymorphisms (SNPs) which were covered by a minimum of four reads in both forward and reverse orientation, four reads calling the allele with at least a phred score of 20, and an allele frequency of 75%. For the detection of insertions and deletion (InDels) we allowed a frequency over 50%.

Deep sequencing was conducted for population diversity analysis with an average genome wide coverage of 200 – 420; SNPs and InDels were called with at least one forward and one reverse read and a phred score >20 for at least 2 reads. All sequences were submitted to the European Nucleotide Read archive, under project accession number ERX5619327.

Three isolates underwent sequencing using the PacBio Sequel II System (Pacific Biosciences). Libraries were prepared with the SMRTbell® Express Template Prep Kit 2·0 according to the manufacturer's instructions. Barcoded overhang adapters for multiplexing were ordered at IDT (Integrated DNA Technologies). During demultiplexing barcodes were filtered for a minimum quality of 50t, which yielded long read sequencing data of an average of 4·4 GB and mean subread length of 9 KB. Long read sequences were *de novo* assembled using PacBio SMRT® Link software version 9 and the "Microbial Assembly" workflow with a set genome size of 4·5 GB with default parameters.

Screening of Rv0678 mutations in clinical samples via the CRyPTIC strain collection

Patient derived Mtbc isolates were collected by CRyPTIC partners throughout 27 countries and analyzed in 14 different laboratories. Strains were selected from the CRyPTIC database for this study if they had matched genotype and phenotype data, a high quality BDQ phenotype, and a mutation in BDQ resistance associated gene (either *atpE*, *Rv1979c*, *pepQ*, *mmpL/S5*, or *Rv0678*). This led to the curation of 179 strains in total.

The full analysis pipeline for CRyPTIC was performed using the Clockwork v0.8.3 pipeline,[3,4] but we outline the key steps here. Sequencing reads were deposited at the European Bioinformatics Institute and run through a bespoke bioinformatics pipeline (publicly available here: https://github.com/iqbal-lab-org/clockwork). In short, reads were filtered against human and other microbial species reads before being mapped to the H37Rv reference genome. Two parallel variant callers (SamTools and Cortex) were used,[5,6] one of which makes high sensitivity SNP calls (SamTools) and one which makes high specificity SNP and indel calls (Cortex). A graph-based adjudication tool (Minos, https://github.com/iqbal-lab-org/minos) was then used to combine these results and create a final set of variants for downstream bioinformatics analysis. All strains were re-genotyped at positions that were variant in at least one CRyPTIC sample, creating a final variant call file with a call for each variant position in the *M. tuberculosis* H37Rv genome.

Phenotyping

In vitro single selected mutants were further analyzed for phenotypic drug susceptibility to both BDQ and CFZ by broth microtiter dilution as a resazurin assay. All mutants were grown in antibiotic free culture media to exponential growth phase (optical density of 0·3-0·8), then diluted to the McFarland standard of 1, with an additional 1:10 dilution, 100 µl of which was seeded in 96-well flat bottom plates (about $1x10^5$ CFU per well). Next 100 µL of antibiotic was added to each well with final concentrations of BDQ as follows: 8, 4, 2, 1, 0·5, 0·25, 0·12, and 0 mg/L; and CFZ: 16, 8, 4, 2, 1, 0·5, 0·25, 0·12, and 0 mg/L. Plates were sealed with permeable tape, and incubated at 37° standing, in sealed plastic boxes. After nine days incubation at 37°, 30µl of resazurin was added to each well. After overnight incubation, fluorescence and absorbance were measure in Biotek plate reader Synergy 2. MIC was determined as the highest concentration which no bacterial growth was detected, either visually or by fluorescence measurement.

For MGIT susceptibility testing, 100µl of frozen bacterial stocks were transferred to Löwenstein-Jensen agar slants and incubated at 37° for three weeks. BACTEC™ MGIT™ 960 SIRE Kit was used and test was carried out according to manufacture instructions. Saline 0.83% solution was used for adjusting bacterial suspension concentration instead of Middlebrook 7H9 broth. Drug concentrations tested were 0·5, 1·0, and 2·0 mg/L for BDQ; 0·5, 1·0, and 2·0 mg/L for CFZ; 0·03, 0·06, and 0·12 mg/L for delamanid; and 0.5, 1·0, and 2·0 mg/L for linezolid. All MGIT tubes which were positive (growth units reached 400) before the antibiotic free growth control were considered resistant. An H37Rv WT strain was not included in this test.

CRyPTIC strains were phenotyped using either the UKMYC5 plate or the updated UKMYC6 plate.[7] Plates were sealed, incubated at 37°C, and read at 14 days. In addition to manual plate readings, all plate images underwent an automated reading using AMyGDA software.[8] Plates without essential agreement for a drug MIC were marked as low quality and sent to a citizen science project (BashTheBug, http://bashthebug.net) for additional verification. Plates with exact agreement between at least two phenotyping methods were marked as high quality. Low (no method agrees) and medium (two methods with essential agreement) quality phenotypes were excluded from this analysis.

All strains included in this study had at least phenotypic data for BDQ. Some strains do not include CFZ data due to missing information, low quality analysis, or removal due to experimental error.

Phenotypic interpretation

Phenotypic interpretation of MIC was determined based on testing method as follows:

- BD-MGIT: susceptible ≤1mg/L, resistant >1mg/L BDQ/CFZ.[9,10]
- UKMYC5/6: susceptible <0.12, borderline 0.12, resistant ≥0.25mg/L BDQ & CFZ, undetermined for wider MIC ranges.
- Resazurin assay: *in vitro* BDQ mutants from this study: resistant ≥1mg/L, genotypic wild-type MIC 0.25-0.5mg/L); in vitro CFZ mutants (this study): resistant >1mg/L, borderline 1 mg/L, (no mutants with lower MIC observed, genotypic wild-type MIC 0. 5-1mg/L).
- Alamar blue/resazurin assay: from literature, resistance defined by primary authors.
- 7H10 plates: resistant ≥0.24mg/L BDQ/CFZ; borderline 0.06-0.12, susceptible <0.06. [11,12]
- 7H11 plates: BDQ resistant ≥ 0.25mg/L,[9] BDQ borderline 0.12, CFZ resistant as defined by primary authors.

Confidence in phenotype interpretation was based on the number of and congruency of strains in the data set, specifically:
high confidence – at least three strains with corresponding phenotypes
medium confidence – at least two strains with correlating phenotypes and a wild-type ancestor
low confidence – two or fewer strains
When a mutation had major discrepancies between number of susceptible and resistant strains these mutations were catalogued as “undetermined”. Due to the potential of *mmpL5* and *mmpS5* variants to

reverse the resistance conferring effect of *Rv0678* variants, strains which harbored also one of these variants were presented individually (appendix 3).

_Rv0678_ variant literature search

An extensive search was performed to include and summarize previously published BDQ and/or CFZ resistant associated mutations from *in vitro*, *in vivo*, and patient derived isolates. We used PubMed, Google, and Google Scholar to search literature published from January 2014 to January 2021. Search criteria included the key "TB", "*Mycobacterium tuberculosis*", "MTB", "bedaquiline", "clofazimine", "treatment", "clinical report", "patient", "MDR-TB", "XDR-TB", "diarylquinoline", and "drug resistance".

Mutations in all resistance associated genes: *Rv0678*, *atpE*, *Rv1979c*, and *pepQ* were included in our final analysis, all variants with low MICs or multiple mutations in the same gene were excluded (appendix 3).

Structural modelling

The crystal structure of Rv0678 (PDB ID: 4NB5) was visualized using UCSF Chimera.[13] To model the DNA-bound form of Rv0678, structural alignment of the wHTH domain was performed using the MatchMaker tool in Chimera with a Needleman-Wunsch algorithm using a BLOSUM-62 matrix and was iteratively pruned until no long atom-pair was > 2 Å resulting in a final average RMSD of 0·81 Å over the 5 guide structures (PDB IDs: 5HSO, 5HSM, 4FX0, 4FX4, 4YIF). All protein stability, protein-protein and protein-DNA interactions were modeled using established mCSM methods with either ligand-bound or DNA-bound Rv0678.[14,15] Mutations that presented both resistant and susceptible phenotypes were treated as resistant during statistical calculations. Screening our resistance catalogue for missense mutations that were resolved in the structure with phenotypes yielded 107 unique missense mutations. mCSM tools are available at http://biosig.unimelb.edu.au/biosig.

Statistical testing of structural features was performed in R, using the Fisher's exact and Wilcoxon rank-sum test on data listed in appendix 2 pp5. SNAP2 scores use machine learning to predict the effect of an amino acid change has on the function of the protein, scores generated for Rv0678 included in appendix 2 pp6 (https://rostlab.org/services/snap2web/). Finally, significant changes in mCSM stability measurements were categorically defined as an absolute change >=1 kcal/mol for the purposes of statistical testing.

Molecular dynamics simulations

The mutated system for these simulations were chosen in order to evaluate the resistant and susceptible phenotype effect on BDQ and CFZ with mutations in the same codon, in order to evaluate the residue type mutation, i.e. affecting the hinge region (not protein folding of DNA binding). All the systems simulated in the present work, including Rv0678-WT, Rv0678-A101E, Rv0678-L40V and Rv0678-L40F, were prepared and simulated using BiKi Life Sciences Software Suite version 1·3·5 of BiKi Technologies s.r.l.[16] Each simulated system consisted of Rv0678 homodimer unit X-ray structure (PDB ID 4NB5) and the mutations were generated using UCSF Chimera software.[13] The Amber14 force field was used in all molecular dynamic simulations performed in BiKi Basic module. TIP3P waters were added to make an orthorhombic box. Adding a suitable number of counter-ions neutralized the overall system. Then, the energy of the whole system was minimized. Four consecutive equilibration steps were then performed: 1) 100ps in the NVT ensemble at 100K with the protein backbone restrained (k=1000 kJ/mol nm^2), 2) 100ps in the NVT ensemble at 200K with the protein backbone similarly restrained, 3) 100ps in the NVT ensemble at 300K with the protein backbone restrained, and 4) 1000-ps in NPT ensemble at 300K with no restraints. For atoms less than 1·1nm apart, electrostatic forces were calculated directly; for atoms further apart electrostatics were calculated using the Particle Mesh Ewald. Van der Waals forces were only calculated for atoms within 1·1 nm of one another. The temperature was held constant using the velocity rescale thermostat, which is a modification of the Berendsen's coupling algorithm. Finally, simulations 100 ns long in the NPT ensemble at 300K were performed for each system. To detect allosteric signal transmission networks across the protein surface, defined as interconnected pocket motions, we carried out the allosteric communication network analysis using the Pocketron module in BiKi Life Sciences Suite version 1·3·5.[16,17]

Authors and members of the Comprehensive Resistance Prediction for Tuberculosis: an International Consortium

(in alphabetical order)

Ivan Barilar[29], Simone Battaglia[1], Emanuele Borroni[1], Angela Pires Brandao[2,3], Alice Brankin[4], Andrea Maurizio Cabibbe[1], Joshua Carter[5], Daniela Maria Cirillo[1], Pauline Claxton[6], David A Clifton[4], Ted Cohen[7], Jorge Coronel[8], Derrick W Crook[4], Viola Dreyer[29], Sarah G Earle[4], Vincent Escuyer[9], Lucilaine Ferrazoli[3], Philip W Fowler[4], George Fu Gao[10], Jennifer Gardy[11], Saheer Gharbia[12], Kelen Teixeira Ghisi[3], Arash Ghodousi[1,13], Ana Luíza Gibertoni Cruz[4], Louis Grandjean[33], Clara Grazian[14], Ramona Groenheit[44], Jennifer L Guthrie[15,16], Wencong He[10], Harald Hoffmann[17,18], Sarah J Hoosdally[4], Martin Hunt[19,4], Zamin Iqbal[19], Nazir Ahmed Ismail[20], Lisa Jarrett[21], Lavania Joseph[20], Ruwen Jou[22], Priti Kambli[23], Rukhsar Khot[23], Jeff Knaggs[19,4], Anastasia Koch[24], Donna Kohlerschmidt[9], Samaneh Kouchaki[4,25], Alexander S Lachapelle[4], Ajit Lalvani[26], Simon Grandjean Lapierre[27], Ian F Laurenson[6], Brice Letcher[19], Wan-Hsuan Lin[22], Chunfa Liu[10], Dongxin Liu[10], Kerri M Malone[19], Ayan Mandal[28], Mikael Mansjö[44], Daniela Matias[21], Graeme Meintjes[24], Flávia de Freitas Mendes[3], Matthias Merker[29], Marina Mihalic[18], James Millard[30], Paolo Miotto[1], Nerges Mistry[28], David Moore[31,8], Kimberlee A Musser[9], Dumisani Ngcamu[20], Hoang Ngoc Nhung[32], Stefan Niemann[29, 48], Kayzad Soli Nilgiriwala[28], Camus Nimmo[33], Nana Okozi[20], Rosangela Siqueira Oliveira[3], Shaheed Vally Omar[20], Nicholas Paton[34], Timothy EA Peto[4], Juliana Maira Watanabe Pinhata[3], Sara Plesnik[18], Zully M Puyen[35], Marie Sylvianne Rabodoarivelo[36], Niaina Rakotosamimanana[36], Paola MV Rancoita[13], Priti Rathod[21], Esther Robinson[21], Gillian Rodger[4], Camilla Rodrigues[23], Timothy C Rodwell[37,38], Aysha Roohi[4], David Santos-Lazaro[35], Sanchi Shah[28], Thomas Andreas Kohl[29], Grace Smith[21,12], Walter Solano[8], Andrea Spitaleri[1,13], Philip Supply[39], Utkarsha Surve[23], Sabira Tahseen[40], Nguyen Thuy Thuong Thuong[32], Guy Thwaites[32,4], Katharina Todt[18], Alberto Trovato[1], Christian Utpatel[29], Annelies Van Rie[41], Srinivasan Vijay[42], Timothy M Walker[4,32], A Sarah Walker[4], Robin Warren[43], Jim Werngren[44], Maria Wijkander[44], Robert J Wilkinson[45,46,26], Daniel J Wilson[4], Penelope Wintringer[19], Yu-Xin Xiao[22], Yang Yang[4], Zhao Yanlin[10], Shen-Yuan Yao[20], Baoli Zhu[47]

Institutions

1 IRCCS San Raffaele Scientific Institute, Milan, Italy
2 Oswaldo Cruz Foundation, Rio de Janeiro, Brazil
3 Institute Adolfo Lutz, São Paulo, Brazil
4 University of Oxford, Oxford, UK
5 Stanford University School of Medicine, Stanford, USA
6 Scottish Mycobacteria Reference Laboratory, Edinburgh, UK
7 Yale School of Public Health, Yale, USA
8 Universidad Peruana Cayetano Heredia, Lima, Perú
9 Wadsworth Center, New York State Department of Health, Albany, USA
10 Chinese Center for Disease Control and Prevention, Beijing, China
11 Bill & Melinda Gates Foundation, Seattle, USA
12 UK Health Security Agency, London, UK
13 Vita-Salute San Raffaele University, Milan, Italy
14 University of New South Wales, Sydney, Australia
15 The University of British Columbia, Vancouver, Canada
16 Public Health Ontario, Toronto, Canada
17 SYNLAB Gauting, Munich, Germany

18 Institute of Microbiology and Laboratory Medicine, IMLred, WHO-SRL Gauting, Germany
19 EMBL-EBI, Hinxton, UK
20 National Institute for Communicable Diseases, Johannesburg, South Africa
21 Public Health England, Birmingham, UK
22 Taiwan Centers for Disease Control, Taipei, Taiwan
23 Hinduja Hospital, Mumbai, India
24 University of Cape Town, Cape Town, South Africa
25 University of Surrey, Guildford, UK
26 Imperial College, London, UK
27 Université de Montréal, Canada
28 The Foundation for Medical Research, Mumbai, India
29 Research Center Borstel, Borstel, Germany
30 Africa Health Research Institute, Durban, South Africa
31 London School of Hygiene and Tropical Medicine, London, UK
32 Oxford University Clinical Research Unit, Ho Chi Minh City, Viet Nam
33 University College London, London, UK
34 National University of Singapore, Singapore
35 Instituto Nacional de Salud, Lima, Perú
36 Institut Pasteur de Madagascar, Antananarivo, Madagascar
37 FIND, Geneva, Switzerland
38 University of California, San Diego, USA
39 Univ. Lille, CNRS, Inserm, CHU Lille, Institut Pasteur de Lille, U1019 - UMR 9017 - CIIL - Center for Infection and Immunity of Lille, F-59000 Lille, France
40 National TB Reference Laboratory, National TB Control Program, Islamabad, Pakistan
41 University of Antwerp, Antwerp, Belgium
42 University of Edinburgh, Edinburgh, UK
43 Stellenbosch University, Cape Town, South Africa
44 Public Health Agency of Sweden, Solna, Sweden
45 Wellcome Centre for Infectious Diseases Research in Africa, Cape Town, South Africa
46 Francis Crick Institute, London, UK
47 Institute of Microbiology, Chinese Academy of Sciences, Beijing, China
48 German Center for Infection Research (DZIF), Hamburg-Lübeck-Borstel-Riems, Germany

References

1 van Soolingen D, Hermans PW, de Haas PE, Soll DR, van Embden JD. Occurrence and stability of insertion sequences in Mycobacterium tuberculosis complex strains: evaluation of an insertion sequence-dependent DNA polymorphism as a tool in the epidemiology of tuberculosis. *Journal of Clinical Microbiology* 1991; **29**: 2578–86.

2 Kohl TA, Utpatel C, Schleusener V, *et al.* MTBseq: a comprehensive pipeline for whole genome sequence analysis of Mycobacterium tuberculosis complex isolates. *PeerJ* 2018; **6**: e5895.

3 Hunt M, Letcher B, Malone KM, *et al.* Minos: variant adjudication and joint genotyping of cohorts of bacterial genomes. *Genome Biol* 2022; **23**: 147.

4 Consortium TCr. A data compendium associating the genomes of 12,289 Mycobacterium tuberculosis isolates with quantitative resistance phenotypes to 13 antibiotics. *PLOS Biology* 2022; **20**: e3001721.

5 Li H, Handsaker B, Wysoker A, *et al.* The Sequence Alignment/Map format and SAMtools. *Bioinformatics* 2009; **25**: 2078–9.

6 Iqbal Z, Caccamo M, Turner I, Flicek P, McVean G. De novo assembly and genotyping of variants using colored de Bruijn graphs. *Nature Genetics* 2012; **44**: 226–32.

7 Rancoita PMV, Cugnata F, Gibertoni Cruz AL, *et al.* Validating a 14-Drug Microtiter Plate Containing Bedaquiline and Delamanid for Large-Scale Research Susceptibility Testing of Mycobacterium tuberculosis. *Antimicrob Agents Chemother* 2018; **62**: e00344-18, /aac/62/9/e00344-18.atom.

8 Fowler PW, Cruz ALG, Hoosdally SJ, *et al.* Automated detection of bacterial growth on 96-well plates for high-throughput drug susceptibility testing of mycobacterium tuberculosis. *Microbiology (United Kingdom)* 2018. DOI:10.1099/mic.0.000733.

9 Technical Report on critical concentrations for drug susceptibility testing of medicines used in the treatment of drug-resistant tuberculosis. https://www.who.int/publications-detail-redirect/WHO-CDS-TB-2018.5 (accessed Nov 9, 2022).

10 Omar SV, Ismail F, Ndjeka N, Kaniga K, Ismail NA. Bedaquiline-Resistant Tuberculosis Associated with Rv0678 Mutations. *New England Journal of Medicine* 2022; **386**: 93–4.

11 Kaniga K, Cirillo DM, Hoffner S, *et al.* A Multilaboratory, Multicountry Study To Determine Bedaquiline MIC Quality Control Ranges for Phenotypic Drug Susceptibility Testing. *J Clin Microbiol* 2016; **54**: 2956–62.

12 Schön T, Juréen P, Chryssanthou E, *et al.* Wild-type distributions of seven oral second-line drugs against Mycobacterium tuberculosis. *Int J Tuberc Lung Dis* 2011; **15**: 502–9.

13 Pettersen EF, Goddard TD, Huang CC, *et al.* UCSF Chimera—A visualization system for exploratory research and analysis. *Journal of Computational Chemistry* 2004; **25**: 1605–12.

14 Pires DEV, Blundell TL, Ascher DB. MCSM-lig: Quantifying the effects of mutations on protein-small molecule affinity in genetic disease and emergence of drug resistance. *Scientific Reports* 2016; **6**: 1–8.

15 Pires DEV, Ascher DB, Blundell TL. MCSM: Predicting the effects of mutations in proteins using graph-based signatures. *Bioinformatics* 2014; **30**: 335–42.

16 Decherchi S, Bottegoni G, Spitaleri A, Rocchia W, Cavalli A. BiKi Life Sciences: A New Suite for Molecular Dynamics and Related Methods in Drug Discovery. *J Chem Inf Model* 2018; **58**: 219–24.

17 La Sala G, Decherchi S, De Vivo M, Rocchia W. Allosteric Communication Networks in Proteins Revealed through Pocket Crosstalk Analysis. *ACS Cent Sci* 2017; **3**: 949–60.

Supplemental Figures

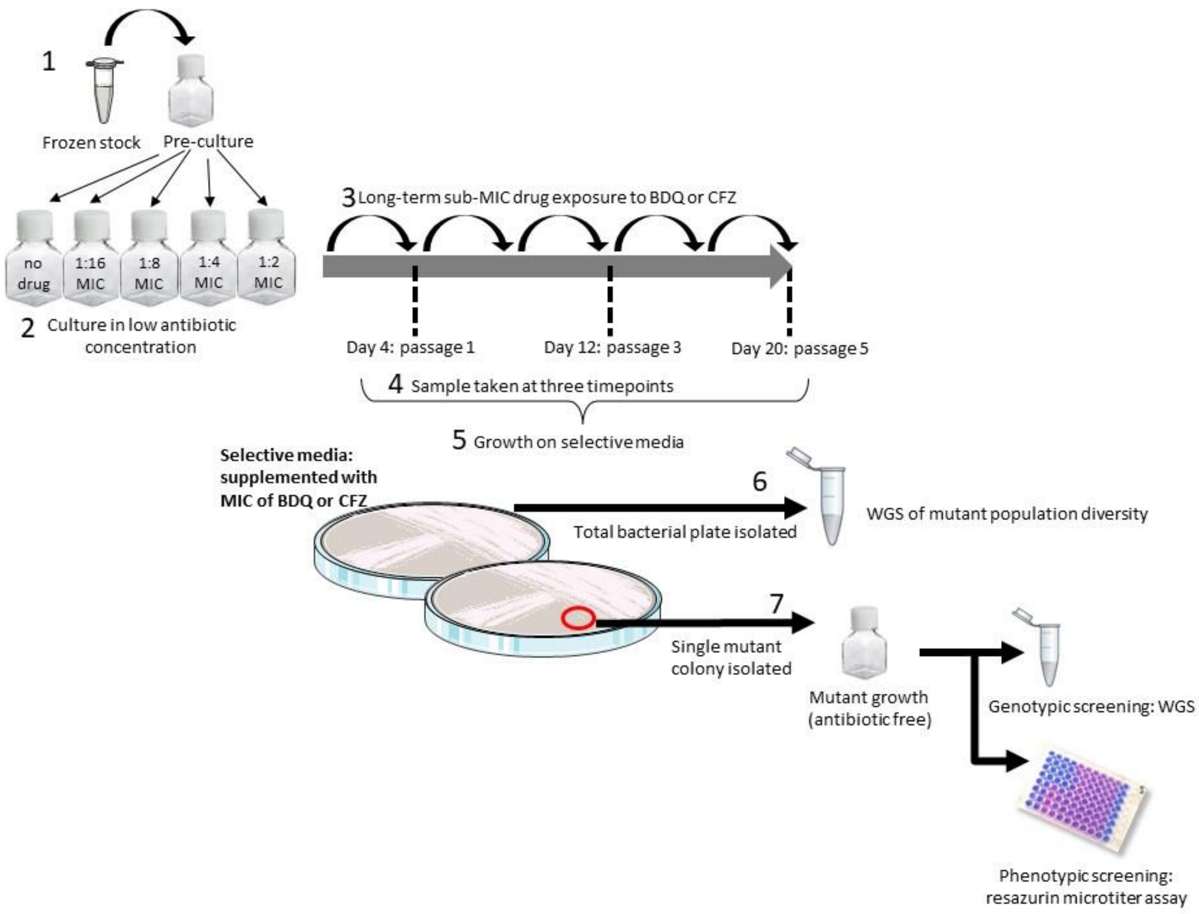

Figure S1: ***In vitro*** **evolutionary experimental design for resistance variant selection, detection and analysis.** (1) First, a pre-culture was started from frozen stocks of *Mycobacterium tuberculosis* complex strain H37Rv, (2) at exponential growth phase bacteria were transferred into new culture bottles and exposed to sub-minimum inhibitory concentrations (MIC) of antibiotics. (3) Bacteria were culture for 20 days including five bacterial passages, (4) cultures were sampled at passages 1, 3, and 5, (5) and grown on selective media plates, supplemented with the MIC of the antibiotic (0·12-0·25 mg/L bedaquiline or 0·25 mg/L clofazimine). (6) After growth on selective media all colonies were pooled and deep sequencing of the heterogenous population was analyzed. (7) Single mutant colonies also were isolated from selective media plates and characterized by whole genome sequencing and MIC tested by resazurin microtiter plate assays.

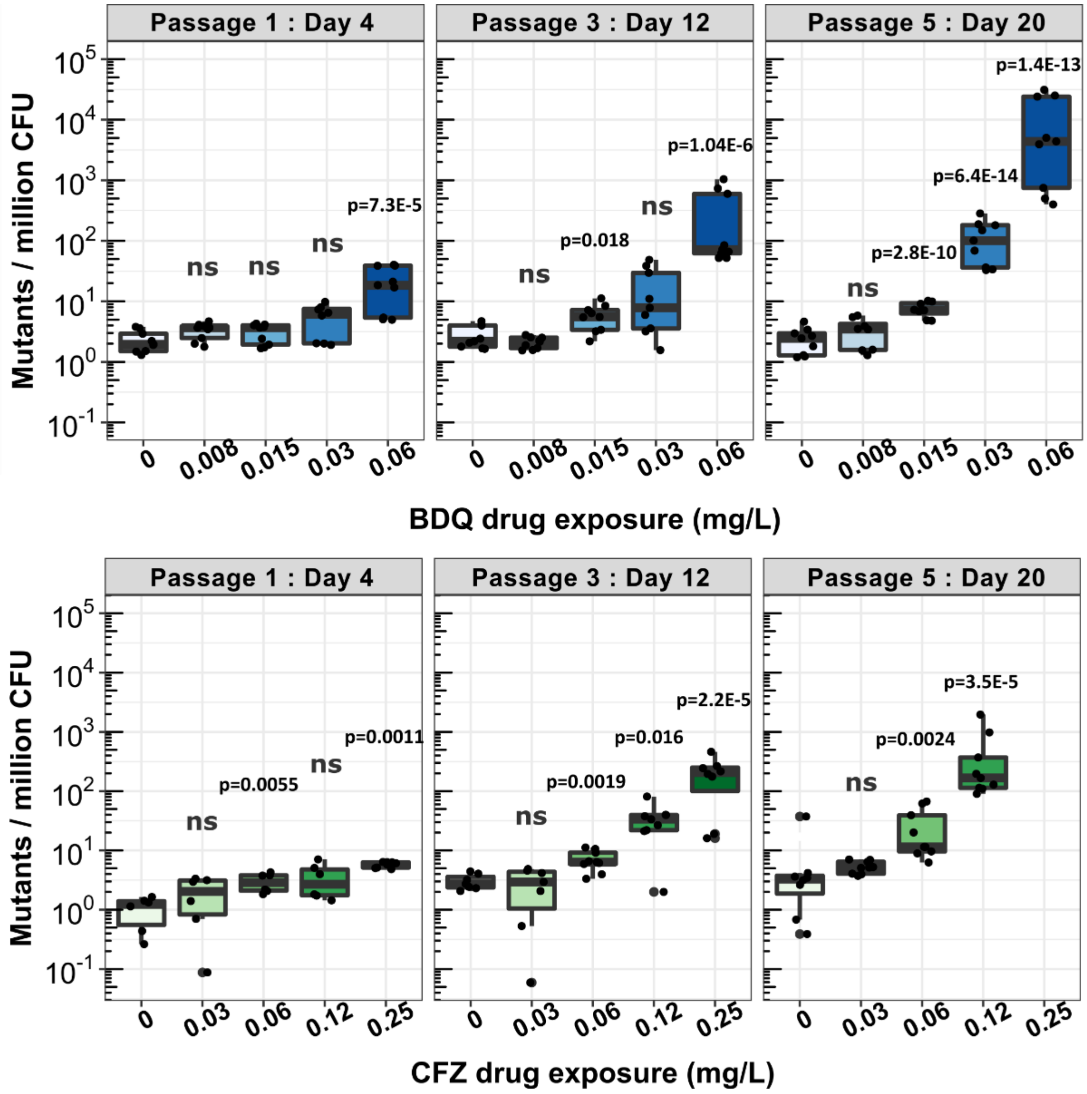

Figure S2: Sub-lethal exposure of antibiotics enriches drug resistant populations in a dose dependent manor. The *Mycobacterium tuberculosis* complex lab strain H37Rv was exposed to four concentrations of bedaquiline (BDQ) (0·06, 0·03, 0·015, and 0·008 mg/L) or clofazimine (0·25, 0·12, 0·06, and 0·03 mg/L), plus an antibiotic free control (0 mg/L BDQ/CFZ), with the highest concentration at 1:2 the minimum inhibitory concentration (MIC), MIC= 0·12mg/L for BDQ and 0·5mg/L for CFZ. The bacteria were exposed to the antibiotic for 20 days, consisting of 5 culture passages. Bacterial samples were evaluated at three timepoints during the experiment, after day 4, day 12, and day 20. Cultures were diluted and plated on 7H11 agar plates, supplemented with and without the MIC of each drug. After 14 to 21 days of growth, colony forming units (CFU) were counted. Mutants per million CFU was calculated by dividing number of mutant CFU/mL by total CFU/mL, then multiplied by 10^6.

Statistics: Three independent experiments were conducted, with 1 biological replicates per experiment, and 3 to 5 technical replicates per biological replicate (9 to 15 values). Statistics was calculated as nonparametric multiple contrast test (Kruskal) with a confidence interval of 95%, p-values between drug exposed and the antibiotic free control.

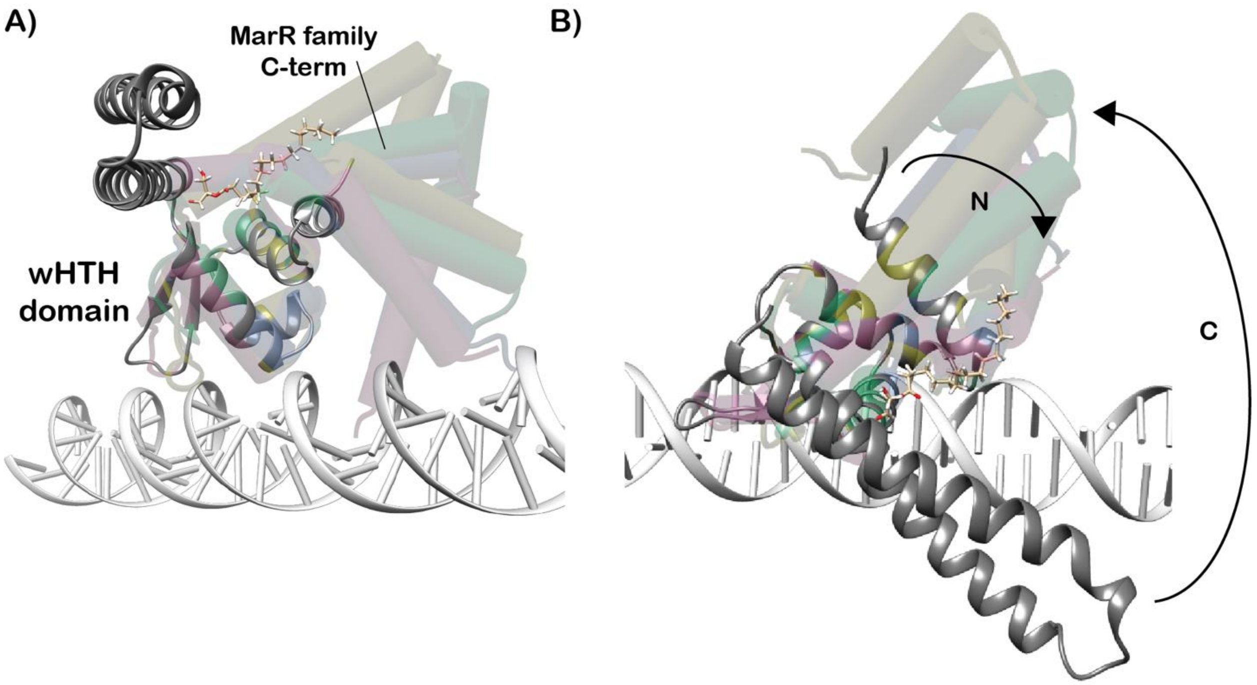

Figure S3: Rv0678 C-terminal domain undergoes conformational change upon DNA binding. (A) Structural alignment of Rv0678 with 5 other MarR-family proteins from Mtbc. Average RMSD across 5 alignments is 0·81 Å. (B) The C-terminal domain conformational change likely involves concerted and counter-directional rotations of the N-terminal helix and C-terminal dimerization domain.

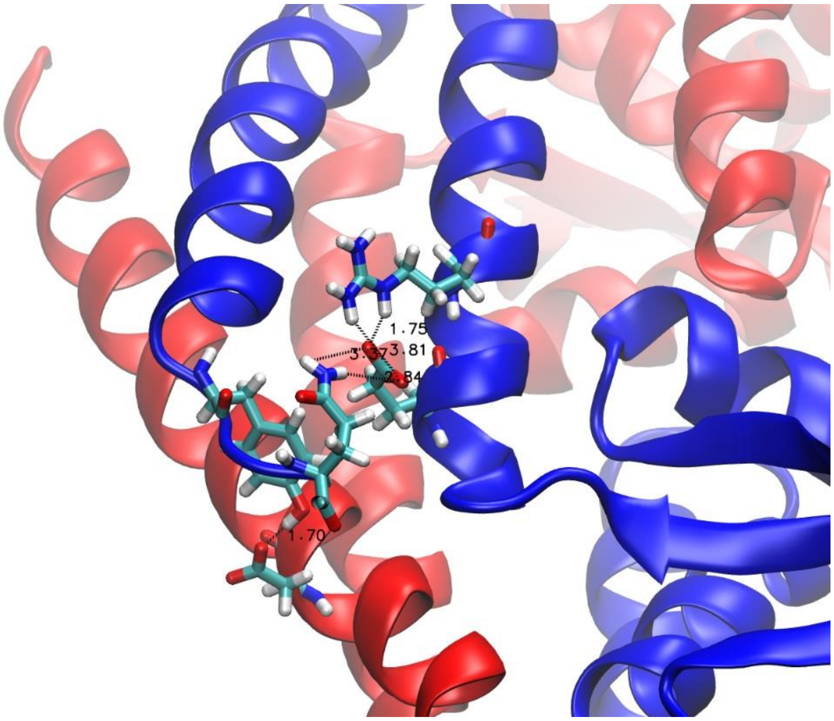

Figure S4. Zoom-in of the persistent interaction along the A101E simulations. The two Rv0678 are shown in red and blue cartoon. The residues involved in the interaction are shown in licorice. In dashed lines are shown the interaction between residues along with the distance in Angstroms unit.

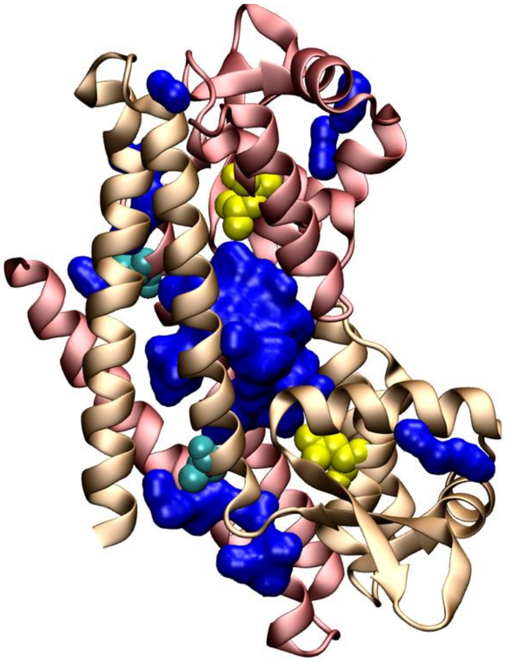

Figure S5: Localization of the main pockets in Rv0678 (4NB5), shown in blue surface. The two monomers are shown in pink and orange cartoon. The residues A101 and L40 are shown in cyan and yellow sphere vdW respectively.

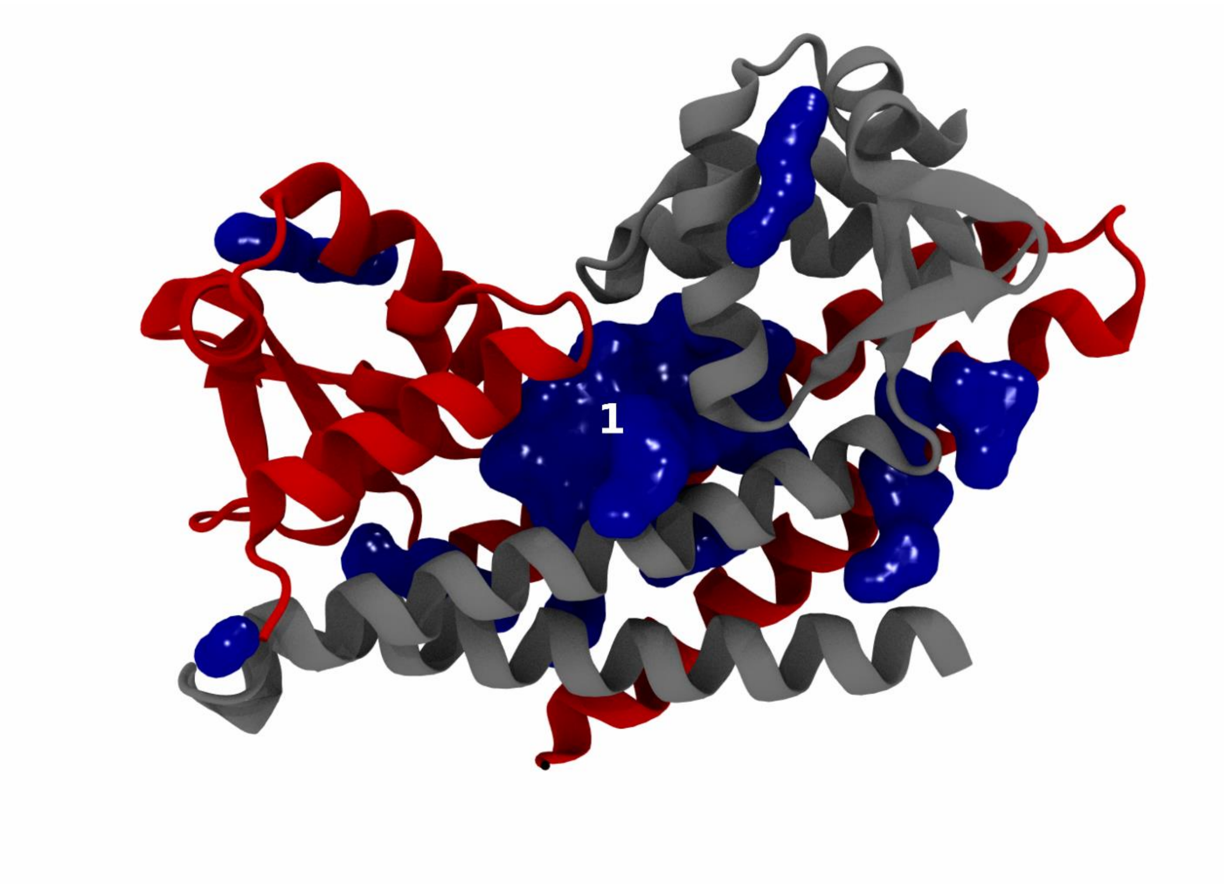

Figure S6: Homodimer pocket communication. Molecular simulations comparing the wild-type and mutant Rv0678 dynamic proteins indicated a common pocket (blue cloud 1).
